# Thermodynamic Properties Investigation of Process Volatile Organic Compounds (VOCs) and Its Transport Impact Factor in Oil Sands Management

**DOI:** 10.3390/nano11030709

**Published:** 2021-03-11

**Authors:** Jing Yuan, Yuyong Sun, Yong Jia, Qianfeng Zhang

**Affiliations:** 1 Department of Civil Engineering, Tongling University, No. 4, Cui Hu Road 1335, Tongling District, Tongling 244000, China; sunyuyong2@126.com; 2 Donadeo Innovation Centre of Engineering, University of Alberta, 9211-116 Street NW, Edmonton, AB T6G 1H9, Canada; 3 CanmetENERGY Devon Research Centre, Natural Resources Canada, 1 Oil Patch Drive, Devon, AB T9G 1A8, Canada; 4 Institute of Molecular Engineering and Applied Chemistry, Anhui University of Technology, Ma’anshan 243002, China; 3421@ahut.edu.cn (Y.J.); zhangqf@ahut.edu.cn (Q.Z.)

**Keywords:** oil sands, thermodynamic, asphalting contents, gas-liquid chromatography (GLC), temperature

## Abstract

This paper presents a new approach for the determination of volatile organic compounds (VOCs) characteristics and their migration influencing factors in oil sands management processes and reveals the relationship between different asphaltene content and different solvents. Specifically, thermodynamic (i.e., partitioning coefficients, Kr, specific retention volume, *V_g_*, the activity coefficients, γ and enthalpy of solution, Δ*H*^0^) and their impact factors are discussed. Gas-liquid chromatography (GLC) experimental measurements were used as the test data. A range of solvents (nC5, iC5, nC6, nC7, and Toluene) has been tested in different asphalt contents (0, 2.56, 9.93, 36.86, 53.67 wt%). There are temperatures in the range of 333.2–393.2 K (with 10 K increase) were conducted, respectively. The dynamics properties of asphalt mixture are calculated, and the relation between dynamics properties of asphalt mixture and absolute temperature, asphalt content and solvent type is discussed. The results show that within the acceptable error range, partitioning coefficients, Kr, specific retention volume, *V_g_*, and enthalpy of solution, Δ*H*_0_ and other thermodynamic properties have a good tendency to predict, they decrease with the increase in asphaltene content and temperature and increase with the increase in solute carbon number.

## 1. Introduction 

During froth treatment and water management processes, oil sands operators try their best to recover as much solvent as possible from the wastewater (i.e., tailings), but a significant amount of light solvent remains are discharged into the environment. Therefore, emissions have a significant impact on air, water, soil pollution and the greenhouse effect [1,2,3]. Therefore, a full understanding of the mechanism of light hydrocarbon release from tailings especially their thermodynamic properties is helpful to find ways to improve the performance of Tailings Solvent Recovery Units (TSRU) [4,5], to reduce the number of light hydrocarbons left in the tailings pond before deposition and to reduce the cost of producing bitumen [6]. In this study, asphaltenes in the form of asphalt or bitumen products are the most valuable component of crude oil, and asphaltene is also considered to be the “heaviest component of petroleum asphalt, insoluble in n-alkanes such as pentane and n-heptane, but soluble in aromatic hydrocarbons such as toluene or xylene.” [6]. The most important is that the highest molecular weight asphaltene components are usually classified as saturated-aromated-resin-asphaltene [7,8]. Asphaltene has complex and highly dispersed components that can form clusters in the nanometer length scale, often defined as “nanoaggregates” [9,10,11,12]. In addition, they can form large aggregates and flocculation, leading to contamination of oil and gas reservoirs, reactors and pipelines, and even serious problems in the transportation and processing of petroleum fluids. It is also difficult to predict when and where these problems will occur during the production process, so it is essential to better understand how its dynamics equilibrium and kinetic processes work to avoid these serious problems [13,14]. The proper dynamics especially the thermodynamic conditions and the choice of the internal structure of TSRU will also affect the kinetics of the asphaltene dilution release process, which plays a crucial role in its performance [15,16]. Dynamics properties of solvents with different proportions of asphaltene are important parameters for modeling and improving the solvent recovery process [17,18]. 

Normally dynamics properties were tested by physical or chemical methods [17,19,20,21]. In recent decades, inverse gas-liquid chromatography (IGC) has become a powerful method to evaluate the properties of various materials [22,23,24]. Therefore, IGC is a useful tool for studying especially thermodynamic properties in this study. The IGC allows access to more thermodynamic parameters (i.e., partitioning coefficients, Kr, specific retention volume, *V_g_*, the activity coefficients, γ and enthalpy of solution, Δ*H*^0^) [25,26,27]. 

For dynamics especially its thermodynamic parameters research, for example, the thermodynamic properties of light hydrocarbons in water/asphalt systems were investigated [20]. Yao et al. further verified the thermodynamic properties of bituminous binder components, conducted molecular dynamics modeling of asphaltenes, and eliminated the uncertainty of some parameter values [28]. Furthermore, the successful application of thermodynamic properties research is limited by its single involved factor. Specifically, Hicks and Young studied the effects of different n-butane, n-pentane, and n-hexane on n-tetracyclane and n-octadecane [29]; However, each measurement is carried out at a separate temperature, so it is almost impossible for these data to be correlated due to the temperature dependence of thermodynamic properties, such as the activity coefficient of low molecular weight solvents at low temperatures [25,26,30]. Besides, most literature reports only data on the activity coefficient of one or more solvents in the high temperature range (one dynamics property) [29,31,32]. Furthermore, Xu and Mundhwa et al. published alkane-solute data at different temperature ranges [33,34]. These literatures were compared with the results of this study. T, carbon number and other single factors have been reported by some researchers in the literature [26,35]. However, there are few studies on asphaltene contents in different solvents and at different temperatures. Therefore, the current research focuses on the use of IGC to study the interactions of different influencing factors with different dynamics properties at different temperatures, from 333.2 K to 393.2 K. In addition, asphalt, an asphaltene derivative, is the material of choice for the experimental samples in this study. Several chemical formulations have been found most effective in dissolving asphaltenes with pure aromatic hydrocarbons such as toluene and xylene. Therefore, in this study, five asphalt samples with different asphaltene contents (0, 2.56, 9.93, 36.86, 53.67 wt%) were used for the fixed phase (solvent/liquid phase) of IGC. In the mobile phase (solute), five light hydrocarbons (2-methylbutane, n-pentane, n-hexane, n-heptane, and toluene) were selected. Finally, the effects of all those different impact factors on different thermodynamic properties such as partitioning coefficients, Kr, specific retention volume, *V_g_*, the activity coefficients, γ and enthalpy of solution, Δ*H*^0^ were compared and evaluated. 

Certain characteristics of oil sand tailings affect the emission rate of VOCs, as well as the residual bitumen in the tailings. It traps volatile organic compounds and slowly releases them into the environment. Thermodynamic parameters are necessary to improve the available technology and predict the distribution of VOCs in solvent recovery process. Therefore, the aim of this research is to start from the basic research to understand its mechanism, to guide the industry to develop new solvent recovery technology. In summary, a better understanding of its thermodynamic properties and their influencing factors will help to optimize or improve solvent recovery and process efficiency, thereby reducing air, water, and soil pollution.

## 2. Research approach 

**Figure 1:**
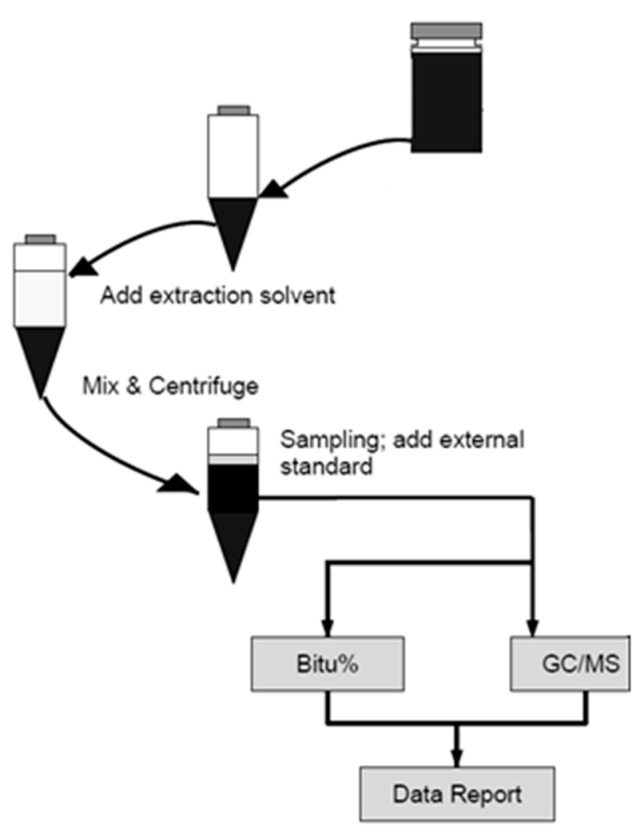
Experimental approach used for the thermodynamic properties determination.

The thermodynamic properties of hydrocarbon vapors in asphalt were mainly tested by two approaches: IGC experimental method and model calculation. A flow chart of the research approach is list in Figure 1.

### 2.1. Materials

Industrial grade (> 99.5%) toluene was used for SARA analysis and solid phase removal. The mass ratios of solvent to asphalt (S/B) were 1.7, 3.5 and 40, respectively, after the dried asphalt was treated with n-pentane. The density of solid asphaltenes ranges from 1100 to 1280 kg/m^3^. After treatment, two fractions were obtained for each S/B: bitumen partially deasphalted and asphaltene-rich deposits of n-pentane evaporated by rotary evaporation. The asphaltene contents were 9.9, 2.6 and 0 wt%, respectively, and the corresponding asphaltene contents in the precipitation were 36.9 wt%, 53.7 wt% and 84.2 wt%, respectively. These partial deasphaltenes and two asphaltene-rich deposits (36.9 and 53.7 wt% asphaltenes) were used as stationary phases of the prepared gas chromatographic column. Table 1 lists five solvents used to prepare stationary phases in gas chromatographic columns, including 2-methylbutane, toluene, pentane, n-hexane, and n-heptane. The purity of n-pentane and n-heptane were 99.5 and 99.7 wt%, respectively. For example, the boiling points of pentane and toluene in Table 1 are 36.1 and 110–111 °C, respectively [36].

Argon and helium gas were purchased from BOC gas. Acid-washed Chromosphere W was pickled and treated with dimethylchlorosilane (DMCS) by the manufacturer of Sigma-Aldrich (Sigma-Aldrich Canada Co., Oakville, ON, Canada).

Five asphalt samples with different asphaltene contents were studied. The high content was 53.7 wt% (% by weight) asphaltene and 36.9 wt% (% by weight) asphaltene, medium content was 9.9 wt% asphaltene, low content was 2.6 wt% and 0 wt% asphaltene. Five different hydrocarbon solvents were then mixed with the five different asphaltenes. For solvents, 2-methyl-butane (iC5), n-pentane (nC5), n-hexane (nC6), toluene, and n-heptane (nC7) are certified by the American chemical society (ACS) and provided by Fisher. The asphaltene content in asphalt was determined by standard pentane precipitation and filtration methods (ASTM D2007-80). In the solvent, the solvent with high solubility and low boiling point is the ideal solvent for deasphaltene, which is the partial deasphaltene or asphaltene rich, precipitates (36.9 and 53.7 wt% asphaltene). The asphaltene content in bitumen was determined by standard pentane precipitation and filtration methods (ASTM D2007-80). A total of thirteen columns with different asphalting percentage were prepared and used for specific retention volume measurements. Table 2 lists the performance of these columns, including the type of bitumen, the asphaltene content, bitumen load in the packing, weight of packing (bitumen coated Chromosorb W), and weight of bitumen in the column (stationary phase). The average molecular weight of each bitumen sample is also included.

### 2.2. Experiment Apparatus and Procedures

Five solvents (nC5, iC5, nC6, nC7 and toluene) with different asphaltene contents (0, 2.56, 9.93, 36.86 and 53.67 wt% asphaltene) were determined at different temperatures (20, 35, 50, 65 and 80 °C). Therefore, a total of 13 experiments were conducted at CanmetENERGY in Devon, Alberta. Peak area and retention time were measured by Agilent 6890 gas chromatography with electronic pressure control (EPC)(Alicat Scientific, Inc., Tucson, AZ, USA), thermal conductivity detector (TCD) and flame ionization detector (FID)( GenTech Scientific LLC, New York, NY, USA). Jour digital flow meter was used to measure the flow at the column outlet. The thermodynamic properties of hydrocarbon vapours in the asphalt were determined by the GLC method. The asphalt as the stationary phase is coated on in a thin layer on Chromsorb W.

Firstly, the asphalt was dissolved in toluene at the calculated concentration to reach the predetermined asphalt load. The toluene asphalt solution was then poured into the pre-weighed Chromsorb W, with the liquid interface is slightly over the solid interface. The Chromsorb W was soaked in the toluene bitumen solution for 24 h. Toluene is then rotated to evaporate slowly at 120 °C and 80 kPa. When most of the toluene is removed and there is no more toluene dripping from the condenser, the pressure is reduced to 6 kPa for half an hour. After the bitumen coated Chromsorb W (g) was placed in a vacuum drying oven at 60 °C and 34 KPa (absolute) for tween-four hours to remove the final toluene. The vacuum oven is under a nitrogen atmosphere. The amount of bitumen coated was determined by measuring the weight loss of a sample on a heating furnace of 2 h at 800 ºC. The column is made of chromatographer-grade stainless steel tube, 2 m long, ¼ inch outside diameter. The tubes were cleaned with deionized water, concentrated nitric acid, deionized water, concentrated ammonium hydroxide, deionized water, and acetone, respectively. A clean stainless-steel tube is then filled with asphalt coated Chromsorb W. one end of the tubing is plugged with a small amount of glass wool and connected to the vacuum pump. The asphalt-coated chrome sand is then slowly poured into the other end of the pipe through a small funnel. During filling, the column is tapped until no more powder can enter the tube. After packing, another glass wool plug is placed on the open end of the tubing. Record the weight of powder. The filled column is coiled and installed under GC conditions at a carrier gas flow rate of 20 mL/min at 100 ºC until the baseline is stable.

Control the carrier gas flow consistently and record the column inlet pressure under each working condition. The dead volume of the packed column was determined by injecting 2 µL argon gas with a 10 uL syringe. Small samples of each investigated hydrocarbon were injected using an automatic sampler at least three times injections to ensure retention time correction. Due to changes in local temperature, air pressure and humidity, the measurements fluctuate slightly. For example, the temperature range is ±0.01 k, and the uncertainty of pressure measurement is 0.01 kPa.

### 2.3. Thermodynamic Methods

To further verify the dynamical model of asphaltene, to eliminate the uncertainty of its parameter values, we use parameters, such as specific retention volume, *V_g_*, solute partition coefficient Kr, and Activity coefficient γ values of the investigated hydrocarbons in bitumen for five different asphalting contents (0, 2.56, 9.93, 21.2, 36.86, 53.67, and 84.2 wt%) to check the thermodynamic properties. It is helpful to discover the change rule of oil sand production, reveal the influence mechanism of oil sand production, and deepen the understanding of the oil sand production process. Initially, *V_g_*^0^ is the volume of the dry carrier gas required to wash and extract the solute sample at 0 °C. The following formula (1–3) is used for the research [37].

(1) $$V_{g}^{0}=\frac{V_{N}}{w_{L}}(\frac{273.15}{T})$$

where,

(2) $$V_{N}=V_{R}^{0}-V_{M}=j(V_{R}-V_{A})=jF_{c}(t_{R}-t_{A})=jF\frac{T}{T_{fm}}(t_{R}-t_{A})$$

(3) $$j=\frac{p_{o}}{\overset{-}{p}}=\frac{3}{2}\left[\frac{{\left(p_{i}/p_{o}\right)}^{2}-1}{{\left(p_{i}/p_{0}\right)}^{3}-1}\right]$$

and, *W_L_* is the mass (g) of the stationary phase (bitumen) in the column, $V_{N}$ is the net retention volume (cm^3^), $V_{R}^{0}$ is the corrected retention volume (cm^3^), $V_{M}$ is the mobile phase volume(cm^3^), $V_{R}$ is the retention volume (cm^3^), *V_a_* is the adjusted retention volume (cm^3^), Fc is the carrier gas flow rate (cm^3^ /min), F is the measured flow rate(cm^3^ /min), T is the column temperature, $T_{fm}$ is the flow meter temperature, *t_R_* is the retention time (min) for a solute; *t_A_* is the column dead time (min); j is the compressibility correction factor; *p_o_* is the outlet pressure (Pa); and *p_i_* is the pressure of the carrier gas at the inlet to the GC column. In this study, based on the results of specific retention volume, $V_{g}^{0}$ from the Equation (1), Partition coefficient, $K_{R}$ can be calculated through the following Equation (4). $\rho_{L}$ is the density of stationary phase (g/cm^3^) and *T* is the column temperature (K) [38,39].

(4) $$K_{R}=\frac{V_{g}^{0}\rho_{L}T}{273.15}$$

Then, according to Equation (4), infinite dilute activity coefficient $\gamma_{p}^{\infty }$ can be calculated through the following Equation (5).

(5) $$\gamma_{p}^{\infty }=\frac{\rho_{L}RT}{K_{R}p_{1}^{0}MW_{L}}$$

where $\rho_{L}$ is the density of stationary phase (g/cm^3^); *MW_L_* is the molecular weight of the stationary phase (g/mol); *R* is the ideal gas constant; $p_{1}^{0}$ is the saturated vapor pressure of the pure solute (Pa) at the column temperature *T* (K).

It can also be seen from equation 4 that the equilibrium constant $K_{R}$ is proportional to the specific retention volume *V_g_*^0^. Therefore, the plot of ln*V_g_*^0^ against 1/T should give the same slope of ln $K_{R}$ versus 1/T. Therefore, then it has been widely used in the following Equation (6) to get the value of enthalpy of solution, Δ*H*^0^ [40,41].

(6) $$\Delta H^{0}=-R\frac{d\ln V_{g}^{0}}{d(1/T)}$$

## 3. Results 

Thirteen tests were prepared for nC5, iC5, nC6, nC7, and Toluene under the different asphalting contents (0, 2.56, 9.93, 36.86, 53.67 and 84.2 wt%) and at different temperature (333.2 K, 343.2 K, 353.2 K, 363.2 K, 373.2 K, 383.2 K, and 393.2 K). Those thirteen tests are used for the specific retention volume *V_g_*^0^ measurements according to Equations (1)–(3). Then, according to the specific retention volume *V_g_*^0^, various thermodynamic properties of the investigated hydrocarbon vapors dissolving into the bitumen, such as partition coefficient Kr and infinite dilution activity coefficient $\gamma_{p}^{\infty }$ are calculated according to Equations (4) and (5). Finally, the results of the measurements of partition coefficient Kr and infinite dilution activity coefficient γ have been used for thermodynamic calculations of enthalpy of solution Δ*H*^0^ according to Equation (6). Those thermodynamic properties especially partitioning coefficient Kr, and activity coefficient γ of the same hydrocarbons in bitumen were discussed in detail. 

### 3.1. Specific Retention Volume 

Uffink et al. describes how macroscopic observations, such as temperature and pressure, relate to microscopic parameters that fluctuate around the mean. It relates thermodynamic quantities (such as volume retention) to microscopic behavior [42]. Therefore, in the study, within the specified temperature range, from 333.2 k to 383.2 k, the obtained specific retention volume values are shown in Table 3 and Figure 1, respectively. Table 3 lists comparisons between referenced data [34] of Specific retention volume $V_{g}^{0}$ and the calculated $V_{g}^{0}$ of investigated hydrocarbons in bitumen under 21.2 % asphalting contents. It could be noted that the measured $V_{g}^{0}$ are in good agreement with the reference data $V_{g}^{0}$ are shown in Table 3. The results also confirmed the reliability of the measurement, which shows that the macroscopic observed values such as temperature and pressure are related to the thermodynamic quantities such as specific retention volume.

**Figure 2:**
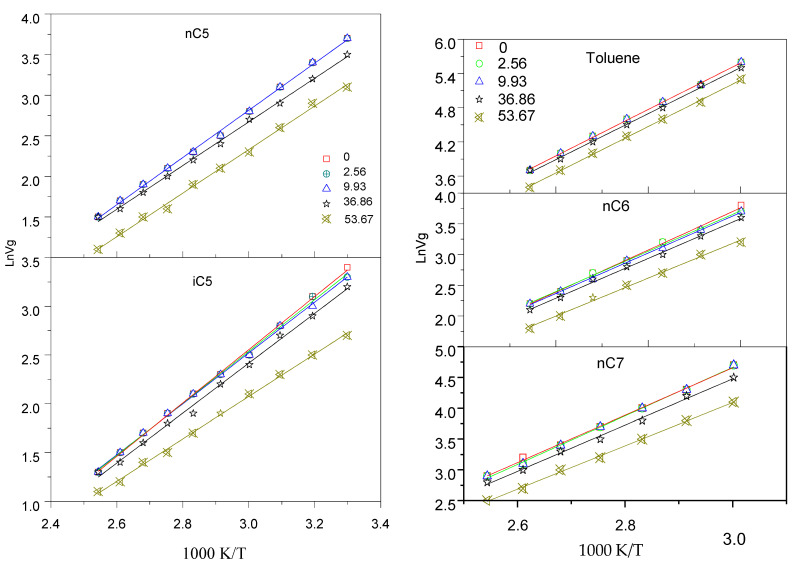
Linear plots for specific retention volume, ln*V_g_*, vs. the inverse of absolute temperature T: nC5, iC5, nC6, nC7, and Toluene under the different asphalting contents (0, 2.56, 9.9336.86, and 53.67 wt%).

The specific retention volume $V_{g}^{0}$ of each i-C5, n-C5, n-C6, n-C7 and toluene compounds under the different asphalting contents (0, 2.56, 9.93, 36.86, 53.67, and 84.2 wt%) were measured and the retention diagrams ln ($V_{g}^{0}$) = f(1/T) were plotted in Figure 2. Straight lines were obtained in Figure 2. Furthermore, for each i-C5, n-C5, n-C7, and toluene it could be noted that the specific retention volume $V_{g}^{0}$ in bitumen that has 2.56 wt% of asphaltene contents is much higher than in the bitumen that has 53.67 wt% asphaltene contents. The bitumen with high asphaltene content has poor retention of hydrocarbons studied. 

At last, As seen in Figure 1, Those hydrocarbons with more carbon atoms have higher $V_{g}^{0}$ values in these adsorbents, whereas the $V_{g}^{0}$ values for light hydrocarbons (≤C5) are almost always less than 100 1/g, which shows that and $V_{g}^{0}$ values increase with the chain length of the solute alkane chain (carbon number).

### 3.2. Partition Coefficient

**Figure 3:**
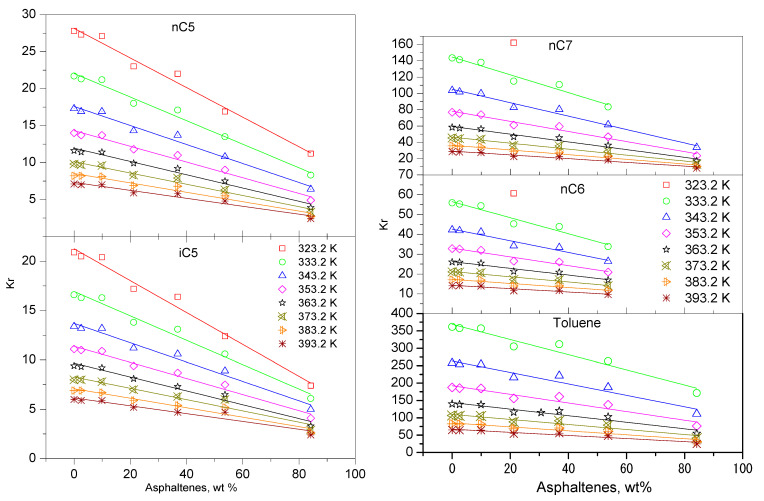
Linear plots for partition coefficient K_R_ vs. the different asphalting contents for different absolute temperature T (333.2 K, 343.2 K, 353.2 K, 363.2 K, 373.2 K, 383.2 K, and 393.2 K) for different dilution: nC5, iC5, nC6, nC7, and Toluene.

It could be noted that in practical case studies, nonpolar or hydrogen bond interactions become important in asphaltene-solvent interactions. In addition, the addition of sufficient non-polar solvents, such as toluene, can inhibit the accumulation of asphaltenes [13]. In fact, these standard chemical reactions, such as partition coefficients, are often used by the asphalt industry: to make them more soluble, alkene substituents, such as pentane, can be added. The results show that increased bond molecular interactions can significantly increase solubility. Therefore, in the study, the partition coefficient K_R_ was calculated from the specific retention volume of Equation (4). The calculated Partition coefficients K_R_ as functions of different asphaltene content (0, 2.56, 9.93, 36.86, and 53.67 wt%) in bitumen for the five hydrocarbons (nC5, iC5, nC6, nC7, and Toluene) are shown in Figure 3. The partition coefficient K_R_ decreases monotonically as the temperature increases. The study also found that in asphalt containing 53.7 wt% asphaltene, the distribution coefficient is usually only 60–70% of those in the bitumen with lower asphaltene contents. 

Similarly, in Figure 3, alkanes and light alkanes have lower distribution coefficients K_R_, while toluene has the highest distribution coefficient K_R_. Additionally, can be seen from Figure 3, the descending order of partition coefficient is: toluene, n-heptane (nC7), n-hexane (nC6), n-pentane (nC5), and 2-methylbutane (iC5). This trend indicates that the role of the molecular geometry of the compounds studied is significant [32].

### 3.3. Infinite Dilution Activity Coefficients

The infinite dilute activity coefficient $\gamma_{p}^{\infty }$ could be calculated in Table 4 according to Equation (5) based on the value of the former partition coefficient K_R_. It can be seen from Table 4 that the value of the infinite dilution activity coefficient $\gamma_{p}^{\infty }$ is related to the content of asphaltene, the type of solute compound (carbon number) and the temperature as well. The saturated vapor pressure of the solute at the column temperature is required. The vapor pressure of the five hydrocarbons at column temperature (Equation (5)) is calculated using the familiar Antoine equation log $p_{1}^{0}$ = A − B/ (t +C), where t is the temperature in degrees Celsius, and A, B and C are known as the Antoine constants, which can be obtained from the literature of Xu et al. [33].

**Figure 4:**
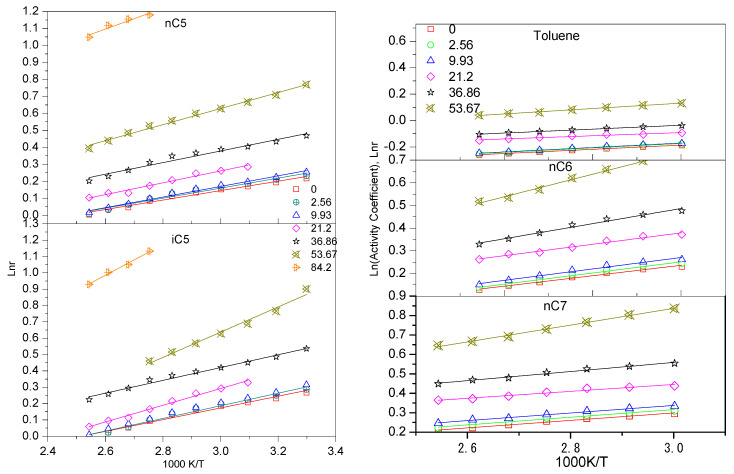
Linear plots for activity coefficients, ln r, vs. the inverse of absolute temperature T: nC5, iC5 nC6, nC7, and Toluene under the different asphalting contents (0, 2.56, 9.93, 21.2, 36.86, 53.67, and 84.2 wt% asphaltene).

The values of the infinite dilute activity coefficient obtained in the specified temperature range and the associated different asphaltene contents for five hydrocarbons are presented in Figure 4, respectively. Figure 3 shows the linear relationship between the natural logarithm of the infinite dilute activity coefficient, ln $\gamma_{p}^{\infty }$, vs the inverse of absolute temperature T. The highest values of infinite dilute activity coefficient $\gamma_{p}^{\infty }$ were obtained for hydrocarbons are with highest asphaltene contents. For the activity coefficient, γ, in bitumen has 18 wt% of asphaltene are much higher than in the bitumen has 84 wt% asphaltene. It is also found that the infinite dilute activity coefficient $\gamma_{p}^{\infty }$ decrease with increasing temperature. The results show that the activity coefficient of infinite dilution increases with the increase in carbon number in a normal alkane. This is because the presence of double and triple bonds in the solute structure significantly reduces the value of the infinite dilution activity coefficient. This is a typical ionic liquid with a weak solution-solvent interaction [7].

The highest values of γ were obtained for normal n-alkanes are for nC7 at the highest asphaltene contents and highest temperature, which was typical of ionic liquid interaction with weak solute-solvent.

### 3.4. Enthalpy of Solution, ΔH_0_

**Figure 5:**
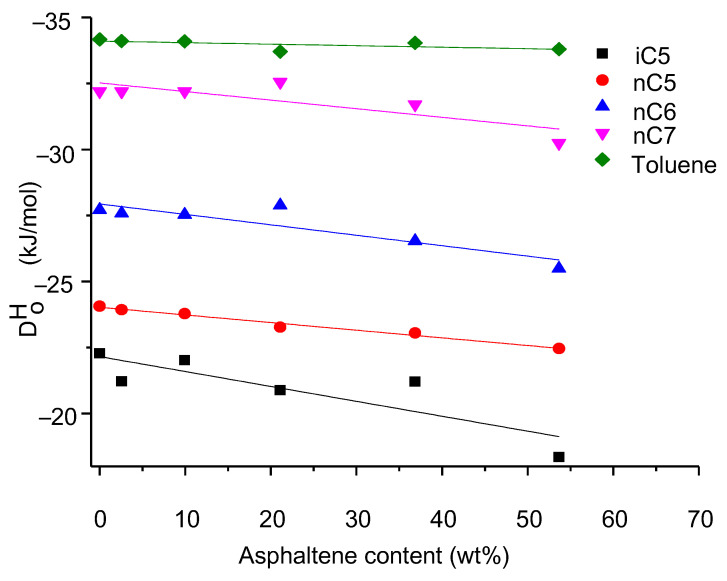
Linear plots for enthalpy of solution, Δ*H*^0^, vs. the different asphalting contents for five hydrocarbons iC5, nC5, nC6, nC7, and Toluene.

Enthalpy of solution Δ*H*_0_ means that in a diatomic molecule AB, the bond energy corresponds to the energy of dissociation in the two atoms A and B, and it indicates the work performed to take them at infinite distance. Like the infinite dilution activity coefficient, the solution enthalpy of a given hydrocarbon compound varies with the change of the carbon number and asphaltene content in the bitumen [7]. Figure 5 shows the comparison of different asphalting contents of five different hydrocarbons (nC5, iC5, nC6, nC7, and toluene). Figure 5 show that the enthalpy of solution Δ*H*_0_ increases with the carbon number in the compound increases. This confirms the occurrence of stronger attractive interactions with the ionic liquid.

## 4. Discussion

**Figure 6:**
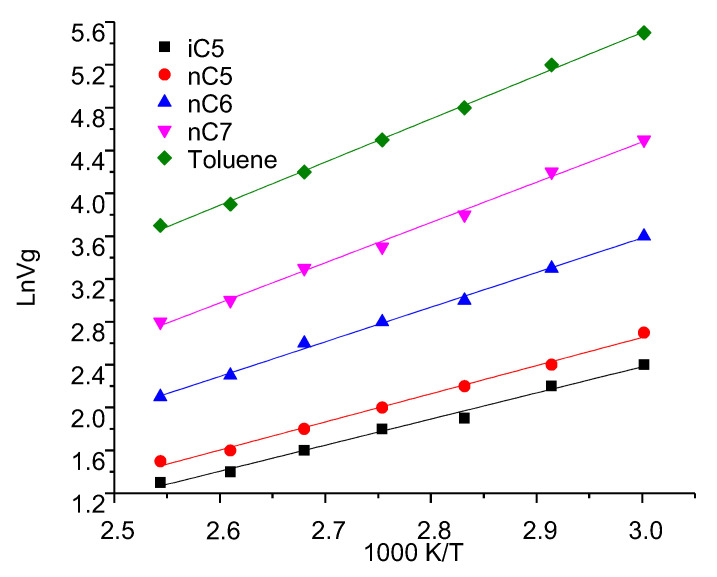
Linear plots for specific retention volume, ln $V_{g}^{}$, vs. the inverse of absolute temperature T for different dilution: nC5, iC5, nC6, nC7, and Toluene under the asphalting contents of 36.86 wt% asphaltene.

**Figure 7:**
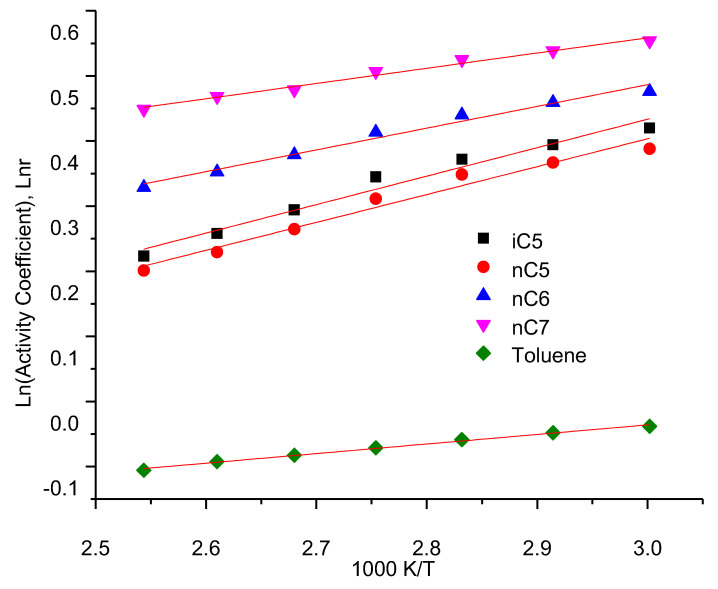
Linear plots for activity coefficients, ln r, vs. the inverse of absolute temperature T: iC5, nC5, nC6, nC7, and Toluene.

Based on the above results, it concluded that a good linear relationship is obtained between the natural logarithm of specific retention volume, $V_{g}^{}$, the partitioning coefficients, Kr, the activity coefficients, γ at infinite dilution, and Enthalpy of solution Δ*H*_0_. Ln $V_{g}^{}$, Ln Kr, LnΔ*H*_0_, Ln γ and the inverse of the absolute temperature, i.e., Ln $V_{g}^{}$, Ln Kr, LnΔ*H*_0_, Ln γ can be expressed like Harned and Robinson [43] as: Ln $V_{g}^{}$ = a + b/(T/K); Ln Kr = a + b/(T/K); and Ln γ = a + b/(T/K) in which a and b are constants (Figure 6). For example, as shown in Figure 6, $V_{g}^{}$ increase with the temperature decrease. Furthermore, as shown in Figure 7, with temperature decrease, Kr, the activity coefficients increase as well.

In this research, $V_{g}^{}$, Kr and Δ*H*^0^ decreases with increasing asphaltene content in the bitumen phase, however, γ will decrease in the opposite way. Furthermore, all the partitioning coefficients, Kr, and specific retention volume, $V_{g}^{}$ the activity coefficients, and enthalpy of solution, γ will decrease with the increasing of temperature and increase with the chain length of the solute alkane chain (carbon number). This phenomenon can be explained by the change of the size ratio of solvent to solute and the interaction between solute and solute. This may also indicate that it is easier to extract these hydrocarbons from the high-asphaltene bitumen found in the TSRU tailings than from the low-asphaltene bitumen from the diluted asphaltene bitumen products [44]. Several reasons might also concur with such outcomes. Firstly, the high temperatures could reduce hydrogen-bonded hydration water, resulting in a decrease in rheological parameters [45].

IGC is a simple, fast, and accurate method to study the thermodynamic interaction parameters and other physicochemical properties of solute in solvent and polymer blends [46,47]. The results show that IGC is a new technique to determine the thermodynamic parameters of asphalt, and the change of asphaltene content and solute has obvious influence on the thermodynamic properties of asphalt. The experimental results are in good agreement with the thermodynamic model, indicating that the solute with high asphaltene content has less retention than that with low asphaltene content [43]. The comparison between the IGC experimental results and the model results shows that when thermodynamic factors are the controlling factors, these process VOCs are easier to be removed from TSRU tailings than traditional asphalt.

## 5. Conclusions

Dynamics parameters of five light hydrocarbons (2-methyl-butane, n-pentane, n-hexane, n-heptane, and toluene) with asphaltene content between 0.0 wt and 53.7% were studied. The values of each thermodynamic parameter in the range of 303.2 K−393.2 K were determined by Inverse gas chromatography (IGC). Firstly, the specific retention volume, $V_{g}^{}$ of hydrocarbons extracted from asphalt with different asphalt contents (0, 2.56, 9.93, 36.86, 53.67, 84.2 wt%) is in good agreement with the experimental data in the literature. Then, the specific retention volume value is used to calculate the thermodynamic properties of hydrocarbon dissolves in asphalt, including partition coefficient, Kr, infinite dilution activity coefficient, γ and enthalpy of solution, Δ*H*^0^.

This study enables us to assess the nature of asphaltene content in the bitumen and the solute compounds’ interactions (hydrogen bonds, solute and solvent interactions). All dynamics parameters (i.e., partitioning coefficients, Kr, specific retention volume, $V_{g}^{}$, the activity coefficients, γ and enthalpy of solution, Δ*H*^0^) of normal alkane increase with increasing chain length. This will help to understand the different behavior of the solute alkanes considering their function and chemical structure, which could be expressed in detail as follows: (1) the results show that the change of asphaltene content and temperature have different effects on the thermodynamic properties of asphalt, and (2) the change of asphaltene content between different light hydrocarbon (carbon number) and asphalt also plays a key role in the control of thermodynamic properties. In summary, a better understanding of its thermodynamic properties and their influencing factors will help to optimize or improve solvent recovery and process efficiency to less VOCs emission, thereby reducing air, water, and soil pollution.

## Figures and Tables

**Table 1:** Properties of isopentane, n-pentane, n-hexane, n-heptane, and toluene using in stationary phases.

	iC5	n-pentane (nC5)	n-hexane (nC6)	n-heptane (nC7)	Toluene
Boiling point °C	27.8–28.2	36.1	68.7	98.42	110–111
Molecular Formula	C_5_H_12_	C_5_H_12_	C_6_H_14_	C_7_H_16_	C_6_H_5_CH_3_
Molecular weight (g/mol)	72.15	72.15	86.18	100.21	92.14
Vapor Pressure (kPa) (at 20 °C)	76.992	57.90	17.60	5.33	2.8
Density @ 25 °C(g/cm^3^)	0.616	0.626	0.655	0.684	0.865

**Table 2:** The properties of different Bitumen/Asphaltenes of different S/B.

Samples for Chromosorb Coating		Bitumen Molecular Weight, g/mol	Weight of Coated Chromosorb, g	Asphaltene Content in Bitumen, wt%	Bitumen Loading, wt%	Bitumen Loading W_L,_ g
Bitumen from different S/B	1.7	442	11.8	9.9	4.4	0.52
	1.7	442	12	9.9	2.6	0.32
	1.7	442	12.1	9.9	10.3	1.25
	3.5	379	11.6	2.6	7.7	0.89
	3.5	379	11.6	2.6	3.1	0.36
	3.5	379	12.2	2.6	13.7	1.67
	40	359	11.6	0	6.0	0.70
	40	359	12	0	2	0.24
	40	359	12.1	0	14.5	1.76
Asphaltenes from different S/B	1.7	1113	12.8	53.7	1.3	0.17
	1.7	1113	13	53.7	2.4	0.31
	3.5	781	12.3	36.9	1.6	0.19
	3.5	781	11.8	36.9	3.0	0.35

**Table 3:** Specific retention volume $V_{g}^{0}$ comparison between references and the calculated $V_{g}^{0}$.

Column Temperature, K	$V_{g}^{0}\;\mathrm{Average}\;(\mathrm{mL}/g)$	$V_{g}^{0}\;*$ (mL/g)	Column Temperature, K	$V_{g}^{0}\;\mathrm{Average}\;(\mathrm{mL}/g)$	$V_{g}^{0}\;*$ (mL/g)
nC5			nC7		
323.15	19.4	18.7	323.15	136.8	
333.15	14.7	14.3	333.15	94.1	92.1
343.15	11.4	11.2	343.15	65.8	
353.15	9.1	8.86	353.15	47.3	46.7
363.15	7.4	7.2	363.15	35.1	34.4
373.15	6.1	5.86	373.15	26.5	26.3
383.15	4.9	3.99	383.15	20.3	
393.15	4.1	3.36	393.15	15.8	15.8
nC6			Toluene		
323.15	51.2		333.15	249.9	245
333.15	37.1	37.1	343.15	171.9	
343.15	27.2		353.15	120.3	120
353.15	20.5	20.7	363.15	87.3	
363.15	16.0	16.3	373.15	64.7	66.2
373.15	12.6	12.6	383.15	48.8	49.1
383.15	10.0		393.15	37.5	38.1
393.15	8.0	8.04			

* Data obtained from reference; Standard uncertainties u are u (*V_g_*^0^) ≤3%, u (T) = 0.5 K.

**Table 4:** Results of infinite dilution activity coefficients ***γ*** for investigated hydrocarbons in bitumen.

Asphaltene Content, wt%	0	2.56	9.93	21.2	36.86	53.67	84.2
Column Temperature, K *							
***γ*** **of iC5**							
303.15	1.31	1.33	1.37		1.71	2.46	
313.15	1.26	1.28	1.31		1.62	2.15	
323.15	1.23	1.25	1.26	1.39	1.57	1.99	3.47
333.15	1.20	1.22	1.23	1.34	1.52	1.87	3.29
343.15	1.18	1.19	1.19	1.30	1.48	1.77	3.13
353.15	1.14	1.15	1.16	1.24	1.45	1.67	3.12
363.15	1.10	1.11	1.11	1.18	1.41	1.58	3.11
373.15	1.05	1.06	1.08	1.12	1.34	1.41	2.86
383.15	1.02	1.02	1.05	1.10	1.29	1.35	2.73
393.15	0.99	1.00	1.01	1.06	1.25	1.27	2.53
***γ* of nC5**							
303.15	1.24	1.26	1.29		1.60	2.16	
313.15	1.21	1.23	1.25		1.54	2.03	
323.15	1.19	1.20	1.22	1.33	1.50	1.95	2.93
333.15	1.17	1.18	1.19	1.30	1.47	1.87	3.04
343.15	1.14	1.16	1.17	1.28	1.44	1.82	3.09
353.15	1.12	1.14	1.14	1.23	1.42	1.74	3.20
363.15	1.09	1.10	1.10	1.19	1.37	1.69	3.25
373.15	1.05	1.07	1.07	1.14	1.30	1.62	3.17
383.15	1.04	1.03	1.05	1.14	1.26	1.55	3.12
393.15	1.00	1.01	1.02	1.11	1.22	1.48	2.85
***γ*** **of nC6**							
333.15	1.26	1.28	1.30	1.45	1.61	2.08	
343.15	1.24	1.26	1.28	1.44	1.58	2.01	
353.15	1.22	1.24	1.27	1.41	1.55	1.93	
363.15	1.20	1.22	1.24	1.37	1.51	1.86	
373.15	1.17	1.19	1.21	1.34	1.46	1.77	
383.15	1.16	1.16	1.19	1.33	1.42	1.71	
393.15	1.14	1.15	1.16	1.30	1.39	1.68	
***γ*** **of nC7**							
333.15	1.34	1.36	1.40	1.55	1.74	2.31	
343.15	1.33	1.35	1.38	1.54	1.71	2.23	4.07
353.15	1.31	1.33	1.36	1.53	1.69	2.15	4.27
363.15	1.29	1.31	1.34	1.50	1.66	2.07	4.37
373.15	1.27	1.29	1.31	1.47	1.61	2.00	4.28
383.15	1.25	1.27	1.30	1.45	1.60	1.95	4.27
393.15	1.23	1.25	1.28	1.44	1.57	1.91	4.12
***γ* of Toluene**							
333.15	0.83	0.84	0.84	0.91	0.96	1.14	1.75
343.15	0.82	0.83	0.83	0.90	0.95	1.12	1.89
353.15	0.81	0.82	0.82	0.90	0.94	1.10	1.99
363.15	0.80	0.81	0.81	0.89	0.93	1.09	2.04
373.15	0.79	0.80	0.80	0.88	0.92	1.06	2.04
383.15	0.78	0.79	0.79	0.87	0.91	1.05	2.05
393.15	0.77	0.78	0.78	0.86	0.90	1.04	2.04

* Standard uncertainties u is u (γ) ≤3%, u (T) = 0.5 K.

## Data Availability

The data presented in this study are available on request from the corresponding author.
